# Correction: Non-thermal plasma modulated l-tyrosine self-assemblies: a potential avenue for fabrication of supramolecular self-assembled biomaterials

**DOI:** 10.1039/d6ra90126c

**Published:** 2026-09-18

**Authors:** Priya Bhatt, Prajakta Sharad Garad, V. V. S. Prasanna Kumari Rayala, P. Radhakrishnanand, Kamatchi Sankaranarayanan

**Affiliations:** a Physical Sciences Division, Institute of Advanced Study in Science and Technology (An Autonomous Institute Under DST, Govt. of India) Vigyan Path, Paschim Boragaon, Garchuk Guwahati Assam 781035 India kamatchi.sankaran@gmail.com; b Academy of Scientific and Innovative Research (AcSIR) Campus Postal Staff College Area, Sector 19, Kamla Nehru Nagar Ghaziabad 201002 Uttar Pradesh India; c Department of Medical Device, National Institute of Pharmaceutical Education and Research SilaKatamur (Halugurisuk), P. O.: Changsari, Dist: Kamrup Guwahati Assam-781101 India; d Department of Pharmaceutical Analysis, National Institute of Pharmaceutical Education and Research SilaKatamur (Halugurisuk), P. O.: Changsari, Dist: Kamrup Guwahati Assam-781101 India

## Abstract

Correction for ‘Non-thermal plasma modulated l-tyrosine self-assemblies: a potential avenue for fabrication of supramolecular self-assembled biomaterials’ by Priya Bhatt *et al.*, *RSC Adv.*, 2024, **14**, 13984–13996, https://doi.org/10.1039/d4ra01891e.

In the original manuscript, the section header for Section 3.2 contains an incorrect term. The authors refer to their analysis as ‘*mass-spectroscopic*’, but the correct term should read ‘*mass spectrometric*’. The section header for Section 3.2 should therefore read ‘3.2 Liquid chromatography-ESI (QQQ) mass-spectrometric analysis’.

In addition, in Section 3.2, the authors assign the peaks in the mass spectra of the protonated control samples of the tyrosine (Tyr)H^+^ state, identifying that the 182.0 *m*/*z* peak corresponds to the protonated tyrosine. Within this section, the authors then state ‘*The other peaks are considered to be arising from the other compounds present in the instrument, categorized as background noise*’. In the absence of definitive peak assignment and appropriate supporting evidence, such signals should not be categorically attributed to background noise. The authors therefore wish to remove this sentence from the original article.

An independent expert has reviewed the corrected information and deemed that a correction is appropriate in this case.

The results and conclusions of this paper are not affected by this correction.

The Royal Society of Chemistry apologises for these errors and any consequent inconvenience to authors and readers.

